# Loss of DJ-1 alleviates microglia-mediated neuroinflammation in Parkinson’s disease via autophagy-lysosomal degradation of NLRP3

**DOI:** 10.3389/fimmu.2025.1656729

**Published:** 2025-09-08

**Authors:** Qianqian Miao, Tiantian Wang, Haoran Wang, Yanxia Yu, Xing Jin

**Affiliations:** ^1^ Department of Pharmacy, The Affiliated Suzhou Hospital of Nanjing Medical University, Suzhou, China; ^2^ Suzhou Municipal Hospital, Suzhou, China; ^3^ Gusu School, Nanjing Medical University, Suzhou, China; ^4^ The Affiliated Suzhou Hospital of Nanjing Medical University, Suzhou, China; ^5^ Nanjing Medical University, Nanjing, China; ^6^ Institute of Neuroscience, State Key Laboratory of Neuroscience, CAS Center for Excellence in Brain Science and Intelligence Technology, Chinese Academy of Sciences, Shanghai, China; ^7^ Department of Clinical Pharmacy, The Affiliated Suzhou Hospital of Nanjing Medical University, Suzhou, China

**Keywords:** Parkinson’s disease, neuroinflammation, DJ-1 (PARK7), NLRP3 inflammasome, microglia

## Abstract

**Objective:**

This study aimed to investigate the role and underlying mechanism of DJ-1 in regulating NLRP3 inflammasome-mediated neuroinflammation during Parkinson’s disease.

**Methods:**

We used LPS to stimulate primary microglia *in vitro* and performed stereotactic injection of LPS into the substantia nigra of mice *in vivo* to investigate changes in DJ-1 expression following inflammatory stimulation. To evaluate the functional effects of DJ-1 on NLRP3 inflammasome activation, we used siRNA to knock down DJ-1 in primary microglia or BMDMs and analyzed downstream inflammatory responses as well as the specificity of this regulation. *In vivo*, we used microglia-specific AAV to selectively silence DJ-1 in the substantia nigra to further evaluate the anti-inflammatory effect of DJ-1 deficiency. To validate the direct interaction between DJ-1 and NLRP3, we performed co-immunoprecipitation and proximity ligation assay. We used the autophagy inhibitor 3-MA and activator rapamycin to investigate how NLRP3 is degraded upon DJ-1 deficiency in CRISPR-Cas9-engineered DJ-1 knockout HEK-293 cells.

**Results:**

DJ-1 were significantly upregulated following LPS or LPS plus ATP stimulation in primary microglia. Similarly, after stereotactic LPS injection into the substantia nigra, we observed a significant upregulation of DJ-1 expression. Knockdown of microglial DJ-1 markedly suppressed NLRP3 inflammasome activation, as evidenced by reduced mature caspase-1 and decreased IL-1β secretion. We confirmed this phenomenon in BMDM and found that DJ-1 knockdown specifically inhibited NLRP3 inflammasome activation, with no effect on NLRC4 or AIM2 inflammasomes. *In vivo*, microglia-specific DJ-1 knockdown significantly attenuated microglial NLRP3 inflammasome activation in the substantia nigra and exerted neuroprotective effects after LPS treatment. Furthermore, DJ-1 was found to directly bind NLRP3 and stabilize its conformation, thereby preventing autophagic degradation.

**Conclusion:**

This study demonstrates that DJ-1 deficiency in microglia inhibits NLRP3-driven inflammation by promoting NLRP3 degradation through the autophagy-lysosome pathway. Future studies should focus on identifying the specific binding sites and structure of DJ-1 with NLRP3, as well as investigating whether inhibiting DJ-1 in microglia could serve as a potential therapeutic target for suppressing neuroinflammation in Parkinson’s disease.

## 1 Introduction

Parkinson’s disease (PD) is a progressive neurodegenerative disorder characterized by the selective loss of dopaminergic neurons in the substantia nigra pars compacta of the midbrain (1, 2). Although the exact pathogenesis of PD remains incompletely understood, accumulating evidence suggests that neuroinflammation plays a central role in the initiation and progression of the disease (3–5). Microglia, the resident immune cells of the central nervous system, are the primary mediators of neuroinflammation (6). In the brains of PD patients, microglia are found to be in a chronically activated state, releasing pro-inflammatory cytokines such as interleukin-1β (IL-1β) and interleukin-18 (IL-18), which contribute to neuronal dysfunction, apoptosis, and necrosis (7). The NOD-like receptor family pyrin domain-containing 3 (NLRP3) inflammasome is a major molecular complex that regulates the maturation and secretion of IL-1β and IL-18 (8). In the brain, NLRP3 is predominantly expressed in microglia and is critically involved in sensing pathogen-associated molecular patterns (PAMPs) or danger-associated molecular patterns (DAMPs) (9). NLRP3 inflammasome activation requires a two-step process: the priming signal, such as lipopolysaccharide (LPS) or inflammatory cytokines, triggers NF-κB-mediated transcription of NLRP3, pro-IL-1β and pro-IL-18, while the activation signal, including ATP and reactive oxygen species (ROS), promotes inflammasome assembly and caspase-1 activation (10, 11). Activated caspase-1 then cleaves cytokine precursors into their biologically active forms and facilitates their release (12).

The activation of the NLRP3 inflammasome is tightly regulated by various endogenous proteins since it contains multiple structural domains that serve as potential binding sites for regulatory proteins (13). For instance, the interaction between NEK7 and NLRP3 is recognized as a critical step for NLRP3 activation (14, 15), whereas the E3 ubiquitin ligase TRIM65 interaction exerts a negative regulatory effect by promoting NLRP3 ubiquitination and degradation (16). To sum up, these interacting partners can either stabilize NLRP3 or promote its degradation, thereby influencing the assembly and activation of the NLRP3 inflammasome (17). Although several regulatory proteins have been identified, the full spectrum of modulators and their mechanisms remains incompletely understood. Identifying additional regulatory proteins—particularly those with inhibitory functions—will be essential for restraining excessive NLRP3 activation in pathological conditions.

DJ-1, encoded by the *PARK7* gene, is a multifunctional protein implicated in the pathogenesis of PD. DJ-1 mutation, such as L166P, could cause early-onset familial PD (18). In neurons, DJ-1 is recognized for its cytoprotective role against oxidative stress, largely through activation of the Nrf2 signaling pathway, which promotes the transcription of antioxidant enzymes (19, 20). Accumulating evidence suggests that DJ-1 may also exert distinct, non-classical functions in glial cells, including the regulation of microglia-mediated inflammatory responses (21). However, although the regulatory effect of DJ-1 on inflammation appears to vary depending on the type of stimulus or disease model, its specific role in LPS plus ATP-induced inflammation, where the NLRP3 inflammasome plays a major part, remains unclear (22–24). A recent study using single-cell RNA sequencing of sorted microglia from DJ-1 knockout mice following LPS stimulation revealed a marked downregulation of multiple inflammation-related genes, including *Nlrp3* and *Il1b*, suggesting a potential pro-inflammatory role for DJ-1 in this context (25). Therefore, clarifying whether DJ-1 acts as an endogenous regulator of NLRP3 and elucidating its role in NLRP3-driven neuroinflammation is of great importance.

In the present study, we found that DJ-1 responds to LPS-induced, NLRP3-driven inflammatory signaling, and that knockdown of DJ-1 exerts anti-inflammatory and neuroprotective effects both *in vitro* and *in vivo*. Furthermore, we demonstrated that DJ-1 can directly bind to NLRP3 and stabilize its conformation. Loss of DJ-1 promoted NLRP3 degradation through the autophagy-lysosome pathway. Collectively, these findings revealed a novel role of DJ-1 in regulating NLRP3 neuroinflammation and a promising therapeutic target for the treatment of PD and other chronic neuroinflammatory diseases.

## 2 Materials and methods

### 2.1 Chemicals and reagents

The following reagents were used: 3-Methyladenine (3-MA, M-9281), Lipopolysaccharide (LPS, L4516), 1-Methyl-4-phenyl-1,2,3,6-tetrahydropyridine (MPTP, M-0896), Nigericin (N-7143), and Poly-L-lysine (P0296) from Sigma-Aldrich; Fetal bovine serum (FBS, 10437028), B27 Supplement (17504044), Neurobasal Medium (21103049), and penicillin-streptomycin (15640055) from Gibco; Lipofectamine 3000 Transfection Reagent (L3000-015), Lipofectamine RNAiMAX Transfection Reagent (13778150), Pierce Protease and Phosphatase Inhibitor Mini Tablets, EDTA-free (A32961) from Thermo Fisher Scientific; Poly (dA:dT)/LyoVec™ (tlrl-patc), and Ultrapure Flagellin (FLA-ST, tlrl-epstfla-5) from Invivogen; DOTAP (HY-112754A) from MedChem Express; Adenosine triphosphate (ATP, A8270) and additional ATP (A8270) from Beijing Solarbio Life Sciences; Rapamycin (53-23-88-9) from Gene Operation.

### 2.2 Antibodies

The primary antibodies used in western blot and confocal microscopy in our present study are listed in **Table 1**.

**Table 1:** List of antibodies used in Western blot and TSA.

Antibodies	RRID	Application	Concentrations	Catalog number	Manufacturer
DJ-1	AB_1310549	WB/TSA	1:1000/1:200	ab76008	Abcam
NLRP3	AB_2490202	WB/TSA	1:1000/1:200	AG-20B-001	AdipoGen
caspase-1	AB_2490248	WB	1:1000	AG-20B-0042	AdipoGen
IL-1β	AB_477105	WB	1:1000	I3767	Sigma
TH	AB_477560	WB/IHC	1:1000/1:500	T1299	Sigma
Bax	AB_2061561	WB	1:1000	50599-2-Ig	Proteintech
BCL-2	AB_1663933	WB	1:1000	BS1511	Bioworld
β-Actin	AB_626630	WB	1:3000	sc-8432	Santa Cruz
GAPDH	AB_627679	WB	1:1000	sc-32233	Santa Cruz
goat anti-mouse IgG-HRP	AB_228307	WB	1:1000	31430	Thermo Fisher
goat anti-rabbit IgG-HRP	AB_631746	WB	1:1000	sc-2004	Santa Cruz
donkey anti-goat IgG-HRP	AB_631728	WB	1:1000	sc-2020	Santa Cruz
IBA-1	AB_839504	TSA	1:500	019-19741	Wako
GFAP	AB_11212597	TSA	1:400	MAB360	Millipore

### 2.3 Experimental animals

Male C57BL/6J mice (2–3 months old, 25–30 g) were obtained from the Animal Core Facility of Nanjing Medical University (Nanjing, China). All animal procedures were approved by the Institutional Animal Care and Use Committee (IACUC) of Nanjing Medical University. Mice were housed under controlled conditions (50-60% humidity, 12-h light/dark cycle) and allowed to acclimate for one week prior to experiments. All mice were specific pathogen-free (SPF).

### 2.4 Primary microglia culture and treatment

Primary microglia were isolated from the brains of postnatal day 1-3 (P1-P3) C57BL/6J mice. Briefly, brains were dissected, meninges and large blood vessels were mechanically removed. Brain tissue was dissociated using 0.25% (w/v) trypsin for 5 min at room temperature, followed by filtration through a 100-µm strainer. After centrifugation (1500 × g, 10 min, RT), cells were plated in poly-L-lysine-precoated flasks containing DMEM/F-12 medium supplemented with 10% (v/v) FBS and 1% (v/v) penicillin-streptomycin. Cultures were maintained at 37°C in a humidified 5% CO_2_ incubator, with medium changes every 5 days. After 12–14 days, microglia were detached by shaking, collected by centrifugation (1500 × g, 10 min, RT), and plated on poly-L-lysine-precoated 24-well plates at a density of 1.9 × 10^5^ cells/ml in complete medium.

Prior to treatment, primary microglia were transfected with DJ-1-specific siRNA or negative control siRNA. Cells were then stimulated with LPS (100 ng/ml) for 8 h followed by ATP (5 mM) for 0.5 h. Cell lysates were analyzed by Quantitative Real-Time PCR (qRT-PCR), western blotting, and co-immunoprecipitation (Co-IP). Cell culture supernatants were assessed for cytokine levels by ELISA.

### 2.5 Primary bone marrow-derived macrophage culture and treatment

BMDMs were isolated from the femurs and tibias of 2-3-month-old male C57BL/6J mice. Bone marrow was flushed with medium. Cells were plated on poly-L-lysine-precoated 24-well plates at 2 × 10^6^ cells/ml in DMEM medium supplemented with 10% (v/v) FBS, 1% (v/v) penicillin-streptomycin, and recombinant mouse M-CSF (0.1‰ w/v, Peprotech, AF-315-03). Cultures were maintained at 37°C in 5% CO_2_. Medium was replaced after 24 h and subsequently every 3 days. BMDMs were used for experiments after 7 days of differentiation.

Before treatment, BMDMs were transfected with si-DJ-1 or control siRNA. Cells were stimulated with LPS (100 ng/ml) 7.5h plus ATP (5 mM) 0.5h, nigericin (10 µM), flagellin (100 ng/ml) 16h, or poly(dA:dT) (2 µg/ml) 14h. Flagellin was incubated with DOTAP at a 1:3 ratio in culture medium for 20 minutes prior to addition to the cells.

Cell lysates were analyzed by western blotting, and supernatants were analyzed by ELISA.

### 2.6 siRNA and plasmid transfection

Adenoviral vectors expressing DJ-1 siRNA (si-DJ-1: sense 5’-ACCUUGCUAGUAGAAUAAACAGU-3’, antisense 5’- ACUGUUUAUUCUACUAGCAAGGU -3’) or control siRNA were obtained from GenePharma. After adherence, primary microglia and BMDMs were transfected with siRNA (200 nM) using Lipofectamine RNAiMAX in serum-free Opti-MEM medium according to the manufacturer’s instructions. Following an 8-h transfection period, the medium was replaced with complete medium, and cells were incubated for a total of 48 h before subsequent treatments.

For NLRP3 plasmid transfection, HEK-293 cells and DJ-1 knockout HEK-293 cells were plated in 6-well plates at 6 × 10^5^ cells/well. Cells were transfected with NLRP3 plasmid using Lipofectamine 3000. After 8 h, the medium was replaced, and cells were treated as indicated. For pharmacological studies, the autophagy inducer rapamycin (10 nM) or the autophagy inhibitor 3-MA (5 mM) was added to the culture medium for 13 h after plasmid transfection.

### 2.7 Primary neuron culture and treatment

Primary cortical neurons were isolated from C57BL/6J mouse embryos at embryonic day 13-16 (E13-E16). Brains were dissected, meninges and blood vessels removed, and tissue dissociated using 0.025% (w/v) trypsin for 30 min at 37°C. The suspension was filtered through a 40-µm strainer. Cells were plated on poly-L-lysine-precoated 24-well or 96-well plates in Neurobasal medium supplemented with 0.5 mM glutamine, 2% (v/v) B27, and 1% (v/v) penicillin-streptomycin. Cultures were maintained at 37°C in 5% CO_2_. Half of the medium was replaced every 3 days. Neurons were used for experiments after 7 days *in vitro*.

Conditioned medium (CM) was collected from treated primary microglia, centrifuged (15,000 × g, 15 min), and the supernatant was mixed 1:1 with fresh Neurobasal medium. Primary neurons in 96-well plates were incubated with this mixture for 6 h prior to the tyramide signal amplification multiplex staining, western blotting and cell viability assay.

### 2.8 Cell viability assay (CCK-8)

Following 6-h incubation with microglial CM, neuronal viability was assessed using the CCK-8 (Bimake, B34304). Briefly, 200 μL CCK-8 reagents diluted at 1:10 in DMEM were added to each well. Plates were incubated under light-protected conditions at 37°C for 2–4 h. Absorbance was measured at 450 nm using a microplate reader (Varioskan Flash, Thermo Fisher). Cell viability was calculated relative to the control wells.

### 2.9 Viral injection and drug treatment

Mice were anesthetized with sodium pentobarbital (40 mg/kg, i.p.) and secured in a stereotaxic frame. To evaluate the neuroprotective role of DJ-1, mice were randomly assigned to six groups (n=6-10/group): AAV-Vector + Saline, AAV-Vector + LPS, AAV-shDJ-1 + Saline, AAV-shDJ-1 + LPS; AAV-Vector + MPTP/p, AAV-shDJ-1 + MPTP/p. shDJ-1 target: sense 5’-ACCUUGCUAGUAGAAUAAACAGU-3’, antisense 5’- ACUGUUUAUUCUACUAGCAAGGU-3’. AAV-shDJ-1-F4/80p-MCS-SV40 PolyA (GenePharma) virus was bilaterally injected (1.5 μL for each side) into the substantia nigra pars compacta (SNc; coordinates relative to bregma: AP: -3.0 mm, ML: ± 1.2 mm, DV: -4.3 mm). Three weeks after viral expression, freshly prepared LPS (0.5 µg) was stereotactically injected at the same site. The surgical site was disinfected with iodophor, and mice recovered on a heating pad. Mice were sacrificed 7 days after LPS injection for subsequent analyses.

For the subacute MPTP/p Parkinson’s disease model, mice received subcutaneous injections of MPTP (25 mg/kg) three weeks after viral expression. MPTP/p was administered once daily for 5 consecutive days and were sacrificed 4 days after the last injection for subsequent analyses.

### 2.10 Behavioral testing

For the locomotor activity test, mice were placed in an activity monitor chamber (50 cm × 50 cm × 40 cm) to acclimatize for 15 min before the start of the test. The track of the mice was recorded for 10 min, and the speed was calculated using the TopScan Realtime Option Version 2.00 behavioral detection system and the supporting camera.

For the rotarod test, the mice were placed on a rod with a diameter of 30 mm and set the program to a low speed of 5 rpm/min, a high speed of 20 rpm/min, and an acceleration time of 2 min. The mice were trained once a day for three days before the formal test so that the mice learned to hold the rod. During the formal test, each mouse was tested three times. The latency to fall was recorded using the Rotarod Analysis System. If the mice could hold for 5 min at any one of the three opportunities, it was recorded as the maximum value of 5 min.

For the pole-climbing test, mice were placed on the top of the wooden pole (diameter 1 cm, height 50 cm, rough surface). The time taken by the mice to turn completely head downward was recorded as t-turn time, and the time taken by the mice to reach the floor with their four paws was recorded as t-total time. Each mouse was accustomed to the apparatus on the day before testing. The test was performed three times to ensure accuracy.

### 2.11 Western blotting and co-immunoprecipitation

Brain tissues or cells were lysed in RIPA buffer (50 mM Tris-HCl pH 7.4, 150 mM NaCl, 1% NP-40) supplemented with protease and phosphatase inhibitors. Protein concentration was determined using a BCA assay kit (P0010, Beyotime Biotechnology). Protein samples (20 µg) were separated by 12% SDS-PAGE and transferred to PVDF membranes (Millipore). Membranes were blocked with 10% non-fat dry milk for 1 h at room temperature and incubated overnight at 4°C with primary antibodies. Membranes were then incubated with appropriate HRP-conjugated secondary antibodies for 1 h at room temperature. Protein bands were visualized using enhanced chemiluminescence (ECL) on an Image Quant LAS 4000 mini (GE Healthcare) and quantified using ImageJ software.

For Co-IP, cells were lysed in RIPA buffer and centrifuged (16,000 × g, 15 min, 4°C). Supernatants containing 500 µg protein were incubated overnight at 4°C with rotation with 10 μL of anti-DJ-1 or anti-NLRP3 antibody. 20 μL Protein A/G agarose beads (sc-2003, Santa Cruz) were added and incubated for 4 h at 4°C with rotation. Beads were washed three times with RIPA buffer. Immunoprecipitated proteins were eluted in SDS-PAGE sample buffer and analyzed by western blotting using the antibodies listed above.

### 2.12 RNA isolation, reverse transcription and quantitative real-time PCR

After treatment, BMDMs or microglia were washed twice with PBS. Total RNA was extracted using Trizol reagent (15596026, Invitrogen) following the manufacturer’s protocol. RNA concentration and purity (A_260_/A_280_ ratio) were determined spectrophotometrically. cDNA was synthesized from 1000 ng RNA using HiScript II Q Select RT SuperMix for qPCR (+gDNA wiper) (R233-01, Vazyme) under the following conditions: reverse transcription at 37°C for 15 min, and enzyme inactivation at 85°C for 5 s. cDNA was diluted 5-fold for qPCR.

qPCR reactions (10 μL) contained 2 μL diluted cDNA, 0.5 μL each of forward and reverse primers (10 µM), 5 μL SYBR Green qPCR Master Mix (P111-01, Vazyme), and 2 μL nuclease-free water. Reactions were performed using the following cycling parameters: 95°C for 10 min, 40 cycles of 95°C for 15 s and 60°C for 1 min, 95°C for 15 s, 60°C for 1 min, 95°C for 15 s.

Primers used were as follows:

*Gapdh*: Forward 5′-CCACTCCAGAAGAAAGACCTAAG-3′,

Reverse 5′-TGGCTGTTGAGGTGCTTATAG-3′.

*Nlrp3*: Forward 5′-CTCAACAGTCGCTACACGCAG-3′,

Reverse 5′-AGCTTAAGGGAACTCATGGGG-3′.

Relative mRNA expression was calculated using the 2^−ΔΔCt^ method with *Gapdh* as the reference gene.

### 2.13 Immunohistochemistry (IHC) and tyramide signal amplification (TSA) multiplex staining

Mice were deeply anesthetized with sodium pentobarbital (1%, 4.5 ml/kg, i.p.) and transcardially perfused with PBS followed by 4% paraformaldehyde (PFA). Brains were post-fixed in 4% PFA for 24–48 h at 4°C, dehydrated in 20% and 30% sucrose-PBS solutions (2–3 days each), embedded in OCT gel, and sectioned coronally at 30 µm thickness.

For IHC (TH staining): Typical brain slices were selected and immersed with 3% hydrogen peroxide (H_2_O_2_) for 15 min to remove endogenous peroxidase. Sections were permeabilized and blocked in PBS containing 0.3% Triton X-100 and 5% BSA for 1.5 h at room temperature. Sections were incubated with anti-TH antibody overnight at 4°C. After PBS washes, sections were incubated with secondary antibodyfor 1.5 h at room temperature, followed by streptavidin-HRP and development with DAB substrate (KGP1045, KeyGEN BioTECH). All the antibodies were diluted in PBS with 1% BSA. Sections were attached on slides, dehydrated through graded alcohols, transparented in xylene, coverslipped with neutral resin, and imaged using an Olympus BX51 microscope.

For TSA multiplex staining: Mounted sections were dried at 37°C, treated with 3% H_2_O_2_ for 10 min at room temperature to remove endogenous peroxidase, and blocked/permeabilized in blocking solution (NEON) for 1.5 h at 37°C. Sections were sequentially incubated with primary antibodies overnight at 4°C, followed by appropriate HRP-conjugated secondary antibodies for 1.5 h at room temperature. TSA fluorophore reagents were applied for 1 min, followed by quenching in distilled water. Antibody complexes were eluted using the provided elution buffer for 30 min at 37°C in the dark before proceeding to the next primary antibody. Nuclei were counterstained with DAPI and photographed by confocal microscope (Lecia SP8).

NLRP3^+^ cell counting and IBA-1^+^ proportion of perikaryon were analysed as previously described (26). NLRP3^+^ cells were quantified within a 0.3 mm² region of interest (ROI) consistently positioned in the SNc of all images. NLRP3^+^ cells, defined by co-localization of a DAPI-stained nucleus with surrounding NLRP3 immunoreactivity, were manually counted with counting software.

To evaluate morphological characteristics of IBA-1^+^ cells within the SNc, the defined SNc region was mapped onto corresponding IBA-1^+^ and DAPI channels. IBA-1^+^ cells exhibiting definitive DAPI^+^ nuclear labeling within the SNc were subjected to quantitative analysis. For each cell, somatic (IBA-1^+^ perikaryon) and nuclear (DAPI^+^) boundaries were manually traced with the ImageJ to generate paired ROIs. The relative somatic area was calculated as: IBA-1^+^ proportion of perikaryon = [(Perikaryon Area - Nuclear Area)/Perikaryon Area]. This metric served as a comparative indicator of perikaryal size alterations across experimental groups.

### 2.14 Proximity ligation assay

The Duolink^®^ PLA kit (DUO92101, Sigma-Aldrich) was used to detect protein-protein interactions. After treatment, cells were washed with PBS, fixed with 4% PFA for 20 min at room temperature, and permeabilized with 0.3% Triton X-100 in PBS for 30 min. Cells were blocked with Duolink^®^ blocking solution for 1 h at 37°C. Primary antibodies (rabbit anti-DJ-1 and mouse anti-NLRP3) diluted in Duolink^®^ antibody diluent were applied and incubated overnight at 4°C. After washing with PBST (PBS containing 0.05% Tween-20), the MINUS and PLUS PLA probes (mixed at a 1:1:3 ratio with antibody diluent) were added and incubated for 1 h at 37°C. The ligation reaction was performed for 30 min at 37°C using the ligation mix (1 μL ligase, 8 μL Duolink^®^ Ligation Buffer and 32 μL deionized water). Amplification was carried out with PLA amplification reagents (8 μL Amplification Buffer, 0.5 μL Polymerase and 32 μL deionized water) at 37°C for 100 min. After PBST washes, F-actin was stained with phalloidin-FITC (US Everbright) for 10–20 min, and nuclei were stained with DAPI for 30 s. Images were acquired using a confocal microscope (Lecia SP8).

### 2.15 High-performance liquid chromatography

Mice were anesthetized and perfused with PBS. Striatal tissues were rapidly dissected on ice, weighed, and homogenized in ice-cold lysis buffer (0.1 M HClO_4_ 0.1 mM Na_2_EDTA; 100 μL/mg tissue) using a pre-chilled homogenizer. Homogenates were centrifuged at 20,000 rpm for 30 min at 4°C. The supernatant was transferred and stored at -80°C until analysis.

Monoamine neurotransmitters and metabolites (dopamine, DA) were quantified using a BAS HPLC-ECD system equipped with an LC-4C electrochemical detector, a C18 reversed-phase column (BAS ODS, 1.0 × 150 mm, 5 µm), a DA-5 chromatography workstation and a fluidic delivery system (+500 mV). The mobile phase consisted of 1.0 mM sodium octanesulfonate, 50 mM NaH_2_PO_4_ 50 mM sodium citrate, 17 mM NaCl, 0.1 mM EDTA, and 4% (v/v) acetonitrile (pH 4.0, adjusted with phosphoric acid). Chromatographic separation was achieved at a flow rate of 0.08 ml/min with a 10 μL injection volume. Quantification was performed using the external standard method.

### 2.16 Cytokine analysis by enzyme-linked immunosorbent assay

Cell culture supernatants were collected after stimulation. Brain tissues were homogenized in ice-cold PBS containing protease inhibitors (10 μL/mg tissue) using a homogenizer, vortexed 10 seconds every 10 min for 30 min on ice, and centrifuged at 16000 g 15 min at 4°C. Supernatants were collected. Concentrations of IL-1β (DY401, R&D Systems) in supernatants and brain homogenates were determined using commercial ELISA kits according to the manufacturers’ protocols. Absorbance was measured at the appropriate wavelength using a microplate reader (Varioskan Flash, Thermo Fisher).

### 2.17 Statistical analysis

All data are presented as mean ± standard error of the mean (SEM). Statistical analyses were performed using GraphPad Prism 10 software. Comparisons between multiple groups were analyzed by Student’s t-test, one-way or two-way analysis of variance (ANOVA), followed by Sidak’s *post-hoc* test. P-value < 0.05 was considered statistically significant. For asymmetric n numbers, Type III sum of squares method were performed before two-way ANOVA.

## 3 Results

### 3.1 DJ-1 expression is upregulated in microglia upon inflammatory stimulation both *in vivo* and *in vitro*

**Figure 1:**
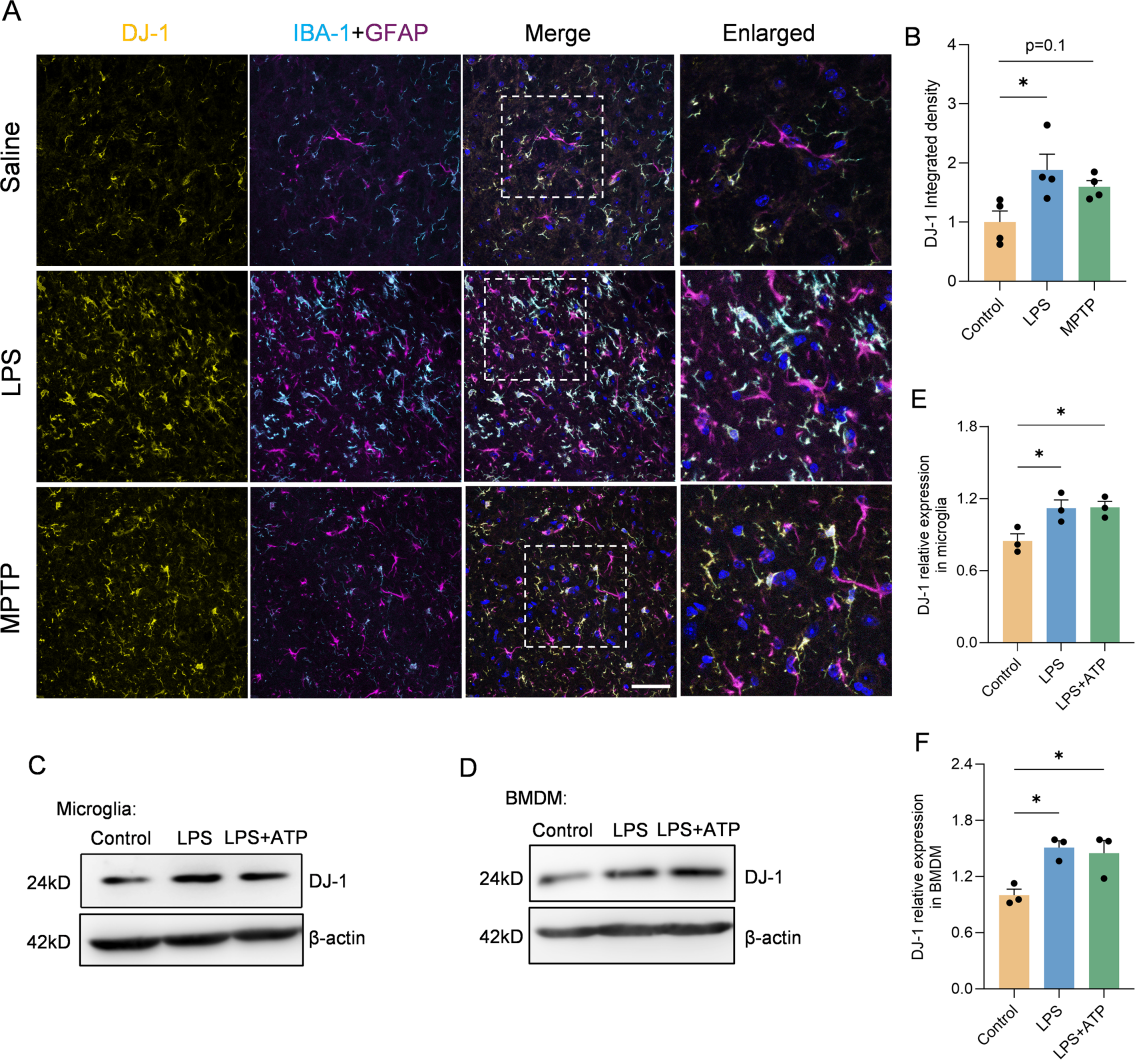
DJ-1 expression was upregulated in LPS induced inflammation both *in vivo* and ex vivo. **(A)** Representative immunofluorescence images of DJ-1 (in yellow), IBA-1 (in cyan), GFAP (in magenta) and hoechst (in blue) in the SNc. The scale bar is 50 µm. **(B)** Quantification of DJ-1 immunofluorescence intensity (n = 4 per group). **(C-F)** Western blotting analysis of DJ-1 in microglia **(C)** and BMDM **(D)** after specific NLRP3 inflammasome stimulation. Quantification of DJ-1 protein level in microglia **(E)** or BMDM **(F)** (n = 3 per group). Data were presented as mean ± SEM. Data were analysed using one-way ANOVA with Sidak’s multiple comparisons test **(B, E, F)**. *P < 0.05.

To demonstrate the association between DJ-1 and inflammatory responses—specifically, that DJ-1 protein expressions are responsive to inflammatory stimuli—we conducted both *in vivo* and *in vitro* experiments. *In vivo*, we established PD models via stereotactic LPS injection into the SNc and via subacute MPTP administration. Immunofluorescence co-labeling of DJ-1 with IBA-1 and GFAP revealed that DJ-1 expression was markedly increased in microglia but not in reactive astrocytes following LPS injection (**Figures 1A, B**). A similar trend was observed in the MPTP model, albeit with a milder inflammatory response (**Figures 1A, B**). Given that NLRP3 inflammasome is the most abundantly expressed inflammasome in microglia, we stimulate primary microglia with the NLRP3 specific activator, i.e., LPS plus ATP. In line with the *in vivo* observations, a marked upregulation of DJ-1 expressions was observed after LPS or LPS plus ATP stimulation (**Figures 1C, E**). Bone marrow-derived macrophages (BMDMs) represent another cell type in which the NLRP3 inflammasome activation pathway is present. Similarly, we found that DJ-1 expressions were significantly upregulated after LPS or LPS plus ATP stimulation (**Figures 1D, F**). These findings suggest that DJ-1 is specifically upregulated in activated microglia under inflammatory conditions in NLRP3 related pathway.

### 3.2 DJ-1 knockdown attenuates NLRP3 inflammasome-mediated inflammatory response in primary microglia and BMDMs

**Figure 2:**
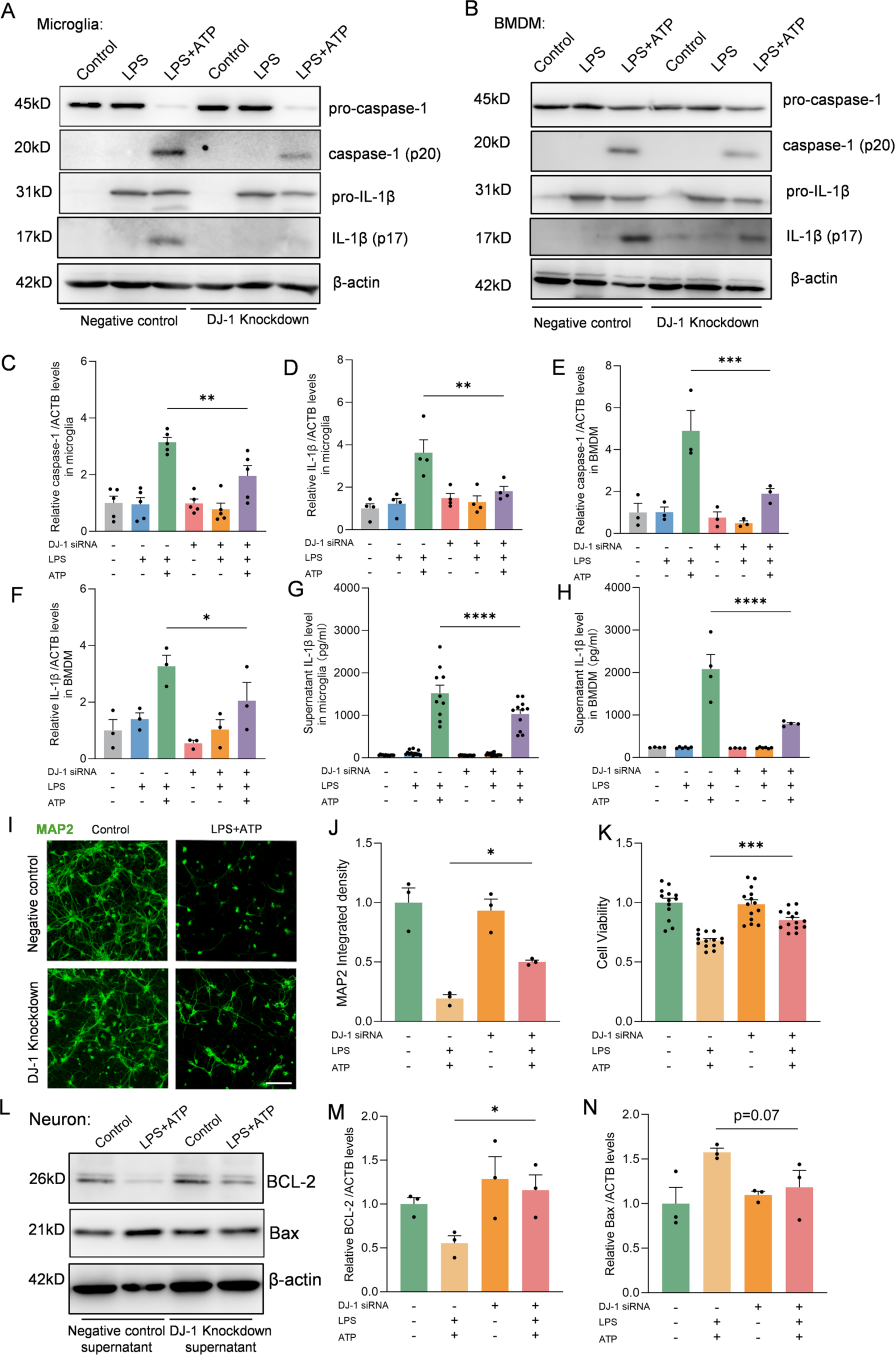
DJ-1 knockdown in microglia and BMDM attenuates NLRP3 related inflammation. **(A-B)** Western blotting analysis of pro-caspase-1, caspase-1, pro-IL-1β and IL-1β in microglia **(A)** and BMDM **(B)** after specific NLRP3 inflammasome stimulation. **(C, D)** Quantification of caspase-1 **(C)** and IL-1β **(D)** protein level in microglia (n ≥ 4 per group). **(E, F)** Quantification of caspase-1 **(E)** and IL-1β **(F)** protein level in BMDM (n = 3 per group). **(G, H)** Elisa analysis of the content of IL-1β in microglia **(G)** and BMDM **(H)** supernatant (n ≥ 4 per group). **(I)** Representative immunofluorescence images of MAP2 (in green) signals in neurons. The scale bar is 10 µm. **(J)** Quantification of MAP2 immunofluorescence relative expression (n = 3 per group). **(K)** Cell viability of neurons (n ≥ 13 per group). **(L)** Western blotting analysis of BCL-2 and Bax. **(M-N)** Quantification of BCL-2 **(M)** and Bax **(N)** protein level in neurons (n = 3 per group). Data were presented as mean ± SEM. Data were analysed using one-way ANOVA with Sidak’s multiple comparisons test **(C-H, J-K, M-N)**. *P<0.05, **P<0.01, ***P<0.001, ****P<0.0001.

To further elucidate the role of DJ-1 during NLRP3 inflammasome activation, we employed siRNA-mediated knockdown of DJ-1 in primary microglia followed by LPS or LPS plus ATP stimulation. DJ-1 expression was efficiently knocked down by siRNA in primary microglia (**Supplementary Figures S1A, C**) and BMDMs (**Supplementary Figures S1B, D**). We found that knockdown of DJ-1 in microglia significantly suppressed NLRP3-mediated inflammatory responses, as evidenced by reduced expression of caspase-1 (p20) and IL-1β (p17) (**Figures 2A, C, D**) as well as attenuated IL-1β secretion into the supernatant detected by Elisa (**Figure 2G**). Similar to primary microglia, knockdown of DJ-1 in BMDMs also significantly suppressed the NLRP3 inflammasome-mediated inflammatory pathway as indicated by decreased caspase-1 (p20) and IL-1β (p17) level (**Figures 2B, E, F**). ELISA of cell supernatants confirmed a significant reduction in IL-1β secretion (**Figure 2H**). Together these data indicate that DJ-1 deficiency dampens NLRP3 inflammasome activation and subsequent inflammatory responses in microglia and BMDMs.

### 3.3 Conditioned medium from DJ-1-deficient microglia reduces neuronal apoptosis

In the brain, activated microglia and the inflammatory cytokines they produce can lead to neuronal apoptosis and axonal degeneration, while suppression of microglia-mediated inflammation can alleviate neuronal damage (27). To further investigate whether DJ-1 knockdown–mediated inhibition of microglial inflammation could alleviate neuronal injury, we treated primary cultured neurons with conditioned medium collected from stimulated control or DJ-1 knockdown microglia. Immunofluorescence staining of MAP2 showed preserved neuronal morphology and reduced neurite fragmentation under the stimuli from DJ-1 knockdown microglia compared to the negative control microglia supernatant (**Figures 2I, J**). Furthermore, CCK-8 assay results showed that primary neurons stimulated with conditioned medium from DJ-1 knockdown microglia exhibited improved cell viability (**Figure 2K**), accompanied by downregulation of the pro-apoptotic protein Bax and significant upregulation of the anti-apoptotic protein BCL-2 (**Figures 2L–N**). These results indicate that knockdown of DJ-1 suppresses microglial inflammatory responses, thereby reducing neuronal apoptosis and axonal degeneration.

### 3.4 DJ-1 specifically regulates the NLRP3 inflammasome but not AIM2 or NLRC4 inflammasomes

**Figure 3:**
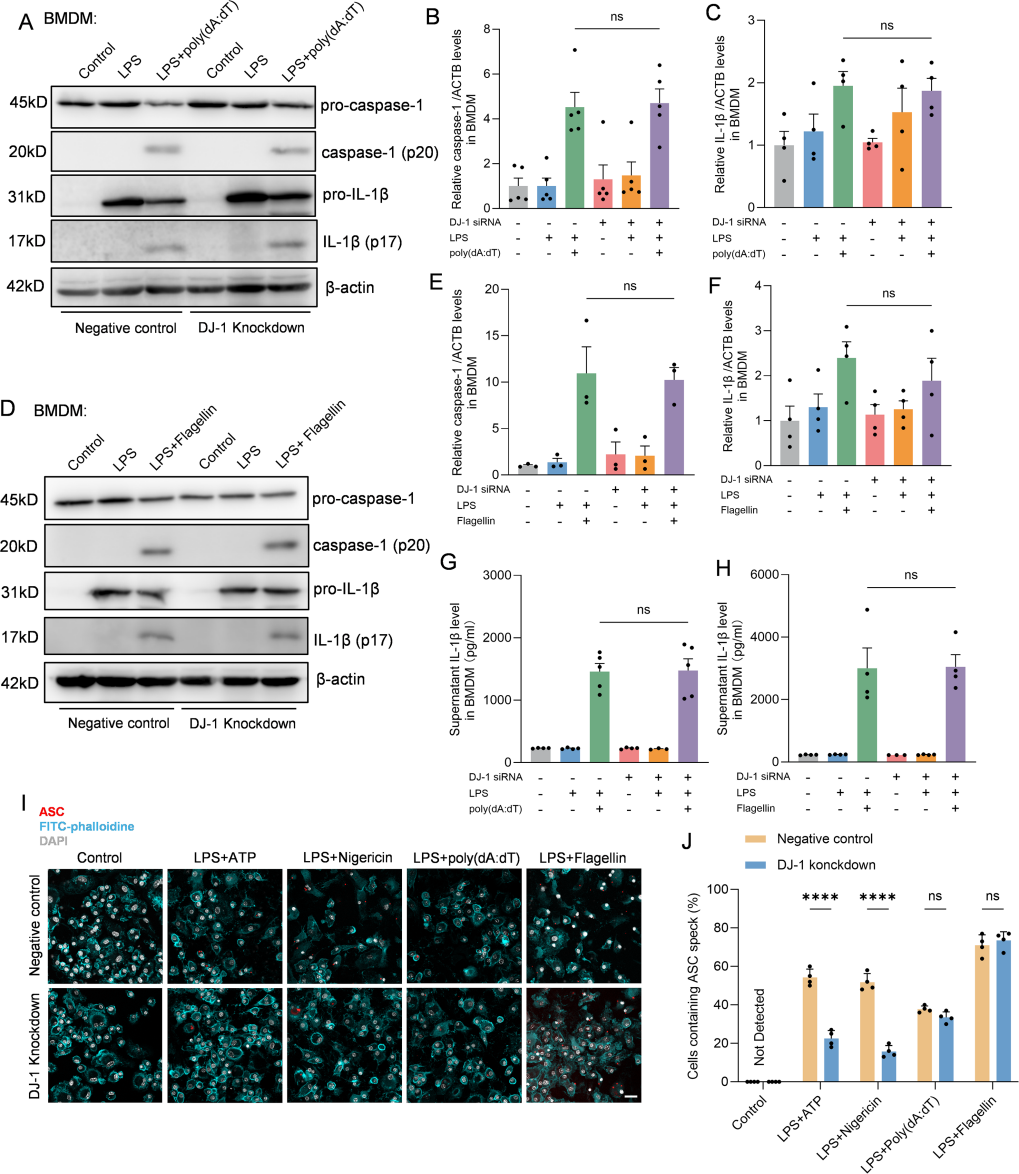
DJ-1 specifically restrains NLRP3 related inflammation but not NLRC4 or AIM2. **(A)** Western blotting analysis of pro-caspase-1, caspase-1, pro-IL-1β and IL-1β after specific AIM2 inflammasome stimulation in BMDM. **(B-C)** Quantification of caspase-1 **(B)** and IL-1β **(C)** protein level in BMDM after specific AIM2 inflammasome stimulation (n ≥ 4 per group). **(D)** Western blotting analysis of pro-caspase-1, caspase-1, pro-IL-1β and IL-1β after specific NLRC4 inflammasome stimulation in BMDM. **(E, F)** Quantification of caspase-1 **(E)** and IL-1β **(F)** protein level in BMDM after specific NLRC4 inflammasome stimulation (n ≥ 3 per group). **(G, H)** Elisa analysis of the content of IL-1β in BMDM supernatant after specific AIM2 **(G)** and NLRC4 **(H)** inflammasome stimulation (n ≥ 3 per group). **(I)** Representative immunofluorescence images of endogenous ASC specks (in red), FITC-phalloidine (in cyan) and DAPI (in grey) in BMDM after specific NLRP3, AIM2 and NLRC4 inflammasome stimulation. The scale bar is 10 µm. **(J)** Quantification of the percentage of cells containing ASC specks in BMDM (n = 4 per group). Data were presented as mean ± SEM. Data were analysed using one-way ANOVA with Sidak’s multiple comparisons test **(B, C, E–H)** and two-way ANOVA with Sidak’s multiple comparisons test **(J)**. Ns, no significance, ****P<0.0001.

In BMDMs, multiple inflammasome activation pathways exist beyond NLRP3, which can be activated by different stimulation combinations but ultimately lead to pro-caspase-1 cleavage and IL-1β secretion following ASC speck formation (28). To further investigate whether the inhibitory effect of DJ-1 on inflammasome activation is specific to the NLRP3 pathway, we stimulated DJ-1 knockdown BMDMs with combinations that specifically activate either the NLRC4 or AIM2 inflammasome. In BMDMs treated with ploy(dA:dT) which could activate the AIM2 inflammasome pathway, we found that no significant changes in neither caspase-1 (p20) nor IL-1β (p17) (**Figures 3A–C, G**). Co-stimulation with LPS and flagellin can activate the NLRC4 inflammasome. We found that DJ-1 knockdown in BMDM had no significant effect on the activation of the NLRC4 inflammasome, as evidenced by unchanged expression levels of caspase-1 and IL-1β (**Figures 3D–F, H**). Besides, immunofluorescence staining of ASC speck formation indicated that the regulatory effect of DJ-1 is specific to NLRP3 inflammasome activation and does not extend to other inflammasome pathways such as AIM2 or NLRC4 (**Figures 3I, J**). Collectively, these results underscore a specific and exclusive regulatory role of DJ-1 in the NLRP3 inflammasome pathway, with no detectable impact on the activation of other inflammasomes such as AIM2 and NLRC4.

### 3.5 DJ-1 knockdown reduces NLRP3 protein levels without affecting its mRNA expression

**Figure 4:**
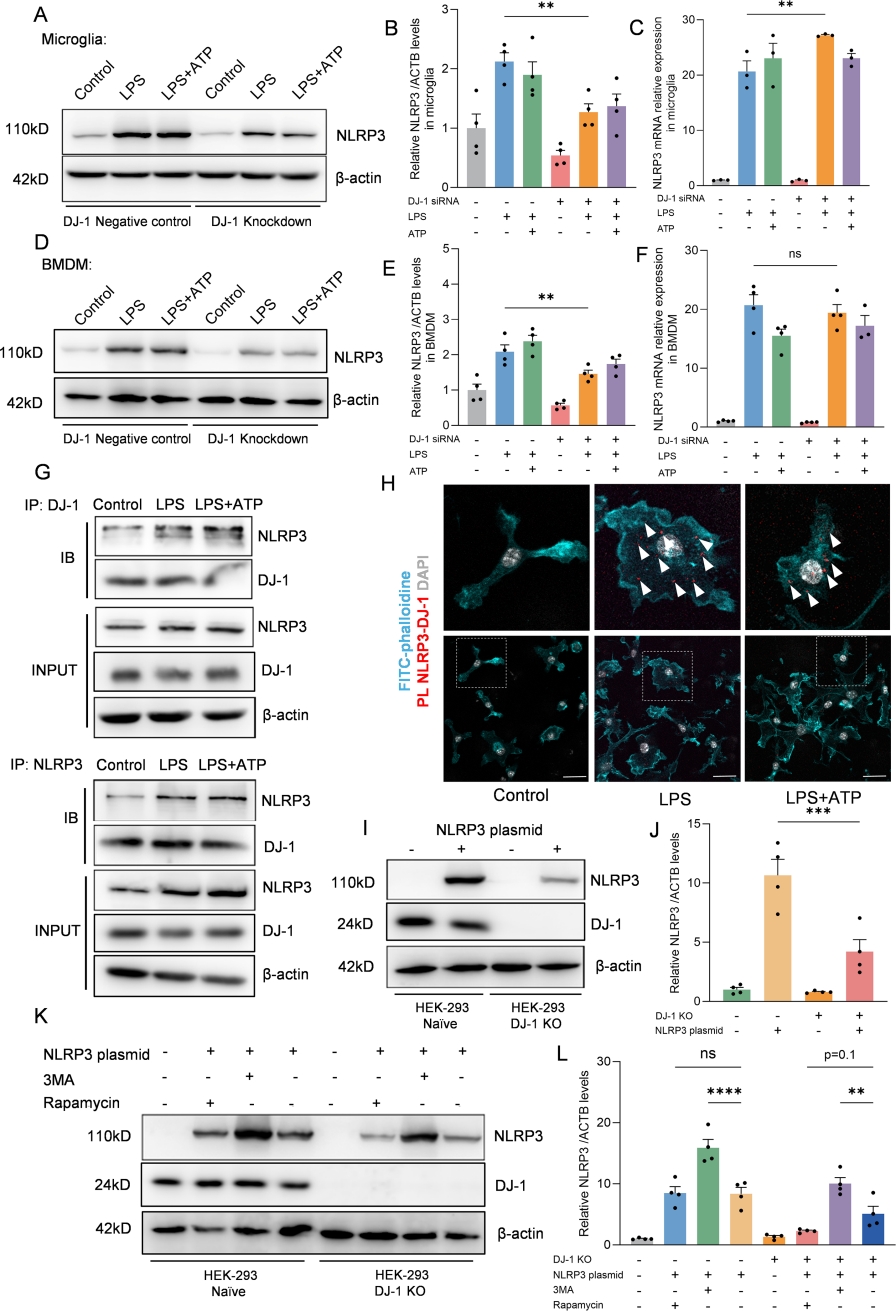
DJ-1 interacts with NLRP3 to inhibit its degradation via the autophagy-lysosome pathway **(A, D)** Western blotting analysis of NLRP3 after specific NLRP3 inflammasome stimulation in microglia **(A)** and BMDM **(D, B, E)** Quantification of NLRP3 protein level in microglia **(B)** and BMDM **(E)** after specific NLRP3 inflammasome stimulation (n = 4 per group). **(C, F)** RT-qPCR analysis of NLRP3 mRNA after specific NLRP3 inflammasome stimulation in microglia **(C)** and BMDM **(F)** (n ≥ 3 per group). **(G)** Microglia were stimulated with 100 ng/ml LPS for 7.5 h and 5 mM ATP for 0.5 h Immunoblotting analysis of NLRP3 protein levels in cell lysates immunoprecipitated with DJ-1 antibody and DJ-1 protein levels in cell lysates immunoprecipitated with NLRP3 antibody. **(H)** Representative images of FITC-phalloidine (in cyan), proximity ligation signals (in red with white arrows) between DJ-1 and NLRP3 and DAPI (in grey). The scale bar is 10 µm. **(I)** Western blotting analysis of NLRP3 and DJ-1 after NLRP3 plasmid transfected into HEK-293 and HEK-293 DJ-1 KO cells. **(J)** Quantification of NLRP3 protein level in HEK-293 and HEK-293 DJ-1 KO cells after NLRP3 plasmid transfection (n = 4 per group). **(K)** Western blotting analysis of NLRP3 and DJ-1 after NLRP3 plasmid-primed HEK-293 and HEK-293 DJ-1 KO cells stimulated with 3MA or rapamycin. **(L)** Quantification of NLRP3 protein level in NLRP3 plasmid-primed HEK-293 and HEK-293 DJ-1 KO cells stimulated with 3MA or rapamycin (n = 4 per group). Data were presented as mean ± SEM. Data were analysed using one-way ANOVA with Sidak’s multiple comparisons test **(B-C, E-F, J, L)**. Ns, no significance, **P<0.01, ***P<0.001, ****P<0.0001.

Given that DJ-1 knockdown selectively impairs NLRP3 inflammasome activation, we next sought to determine whether this regulatory effect involves direct modulation of the NLRP3 protein expression levels. In both primary microglia and BMDMs, DJ-1 knockdown led to a significant reduction in NLRP3 protein levels after LPS or LPS plus ATP stimulation (**Figures 4A, B, D, E**). However, mRNA levels of NLRP3 remained unchanged (**Figures 4C, F**). These results suggest that DJ-1 knockdown does not affect the transcriptional activity of NLRP3 but instead regulates its protein stability through a post-transcriptional mechanism. Specifically, we hypothesize that DJ-1 may stabilize the NLRP3 protein structure and prevent its degradation. Therefore, we hypothesize that DJ-1 may function as a stabilizing factor for NLRP3 by directly interacting with the NLRP3 protein. Co-immunoprecipitation assays in primary microglia demonstrated a direct interaction between DJ-1 and NLRP3 proteins (**Figure 4G**). Furthermore, Proximity ligation assays (PLA) revealed that DJ-1 and NLRP3 are co-localized in the cytoplasm of microglial cells upon inflammatory stimulation, further supporting the possibility of a functional interaction (**Figure 4H**).

To further investigate the relationship between DJ-1 and NLRP3, we generated DJ-1 knockout HEK-293 cells using CRISPR-Cas9 technology. We then transfected equal amounts of NLRP3 plasmid into both DJ-1 knockout and control cells. The results showed that the protein level of NLRP3 was significantly reduced in DJ-1-deficient HEK-293 cells, consistent with the observations in primary microglia and BMDMs (**Figures 4I, J**). NLRP3 harbors multiple protein-interacting domains on its surface and has been reported to bind to several regulatory proteins such as NEK7 (29) or TRIM65 (16) which contribute to stabilizing its conformation. We hypothesize that DJ-1 may exert a similar stabilizing function as these proteins. Our results demonstrated that inhibition of autophagy with 3-MA led to a significant increase in NLRP3 protein levels in both wild-type and DJ-1 knockout HEK-293 cells, confirming that NLRP3 is degraded, at least in part, through the autophagy-lysosome pathway. Furthermore, due to the absence of DJ-1, rapamycin-induced autophagy caused a greater reduction in NLRP3 levels in DJ-1 knockout cells than in wild-type cells (**Figures 4K, L**). These findings support the notion that DJ-1 stabilizes NLRP3 by protecting it from autophagic degradation, likely through maintaining its proper conformational structure. In summary, we found that DJ-1 binds directly to the NLRP3 protein through a post-transcriptional mechanism, stabilizing its conformation and protecting it from autophagy–lysosome-mediated degradation. In the absence of DJ-1, this protective effect is lost, resulting in enhanced degradation of NLRP3 and subsequent downregulation of the inflammatory signaling pathway.

### 3.6 Stereotaxic knockdown of DJ-1 in SNc microglia attenuates neuroinflammation and TH^+^ neuron loss *in vivo*

**Figure 5:**
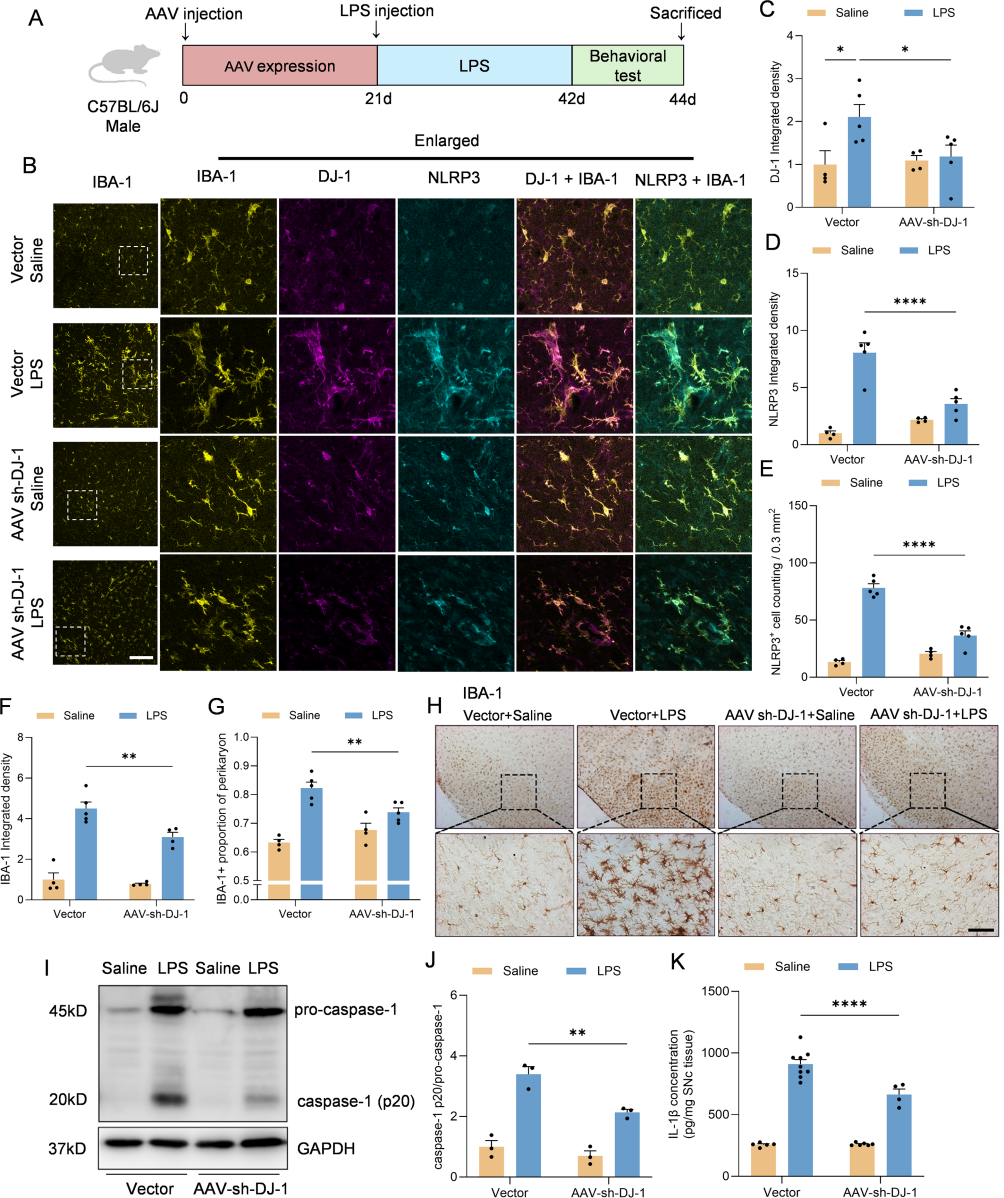
Microglia-specific DJ-1 knockdown attenuates LPS-induced microglial activation and inflammatory responses **(A)** Schematic representation of the experimental flow. C57BL/6J mice were injected with either AAV-Vector or AAV-shDJ-1 and after 21 days were treated with LPS. **(B)** Representative immunofluorescence images of DJ-1 (in magenta), IBA-1 (in yellow) and NLRP3 (in cyan). The scale bars are 100 μm and 25μm. **(C, D)** Quantification of DJ-1 **(C)** and NLRP3 **(D)** immunofluorescence relative expression (n ≥ 4 per group). **(E)** Quantification of NLRP3+ cell counting within 0.3 mm2 (n ≥ 4 per group). **(F, G)** Quantification of IBA-1 immunofluorescence relative expression **(F)** and IBA-1^+^ proportion of perikaryon **(G)** (n ≥ 4 per group). **(H)** Microglia activation was examined by IHC detection of IBA-1. The scale bar is 200 μm. **(I)** Western blotting analysis of pro-caspase-1, caspase-1 and pro-IL-1β in the midbrain homogenate from the indicated mice. **(J)** Quantification of caspase-1 protein level in the midbrain homogenate supernatant (n = 3 per group). **(K)** Elisa analysis of the content of IL-1β in the midbrain homogenate supernatant (n ≥4 per group). Data were presented as mean ± SEM. Data were analysed using two-way ANOVA with Sidak’s multiple comparisons test **(C-G, J)**. For **(K)**, the data were log_10_-transformed to eliminate heteroscedasticity, and subsequently analyzed using two-way ANOVA. *P<0.05, **P<0.01, ****P<0.0001.

Given that microglial activation and NLRP3 inflammasome-mediated neuroinflammation are critical pathological mechanisms in Parkinson’s disease (PD), we employed an *in vivo* model involving stereotactic injection of LPS into the substantia nigra to induce PD-like pathology (**Figure 5A**). To specifically knock down DJ-1 in microglia, we used an adeno-associated virus (AAV) vector expressing DJ-1 shRNA under the control of the microglia-specific promoter F4/80, and we confirmed that the AAV-mediated knockdown effectively reduced DJ-1 expression (**Figures B, C**). The AAV was stereotactically injected into the substantia nigra pars compacta (SNc), and after three weeks for viral expression, we performed a second stereotactic injection of LPS at the same site. Immunofluorescence analysis revealed that, following stereotactic injection of LPS into the SNc, mice with microglia-specific DJ-1 knockdown exhibited a markedly attenuated microglial inflammatory response, along with significantly reduced NLRP3 protein expression and reduced quantity of NLRP3^+^ cells (**Figures 5B, D, E**). IBA-1 integrated density level as well as proportion of perikaryon IBA-1 positive cells were attenuated after AAV-sh-DJ-1 injection (**Figures B, F, G**). Immunohistochemical staining for IBA-1 further confirmed that microglial activation was markedly reduced in mice with microglia-specific DJ-1 knockdown (**Figure 5H**). In addition, following LPS administration, the midbrain tissue of DJ-1-deficient mice exhibited significantly lower caspase-1 levels compared to control mice (**Figures 5I, J**). Consistently, ELISA analysis revealed a significant reduction in IL-1β levels in the midbrain (**Figure 5K**).

**Figure 6:**
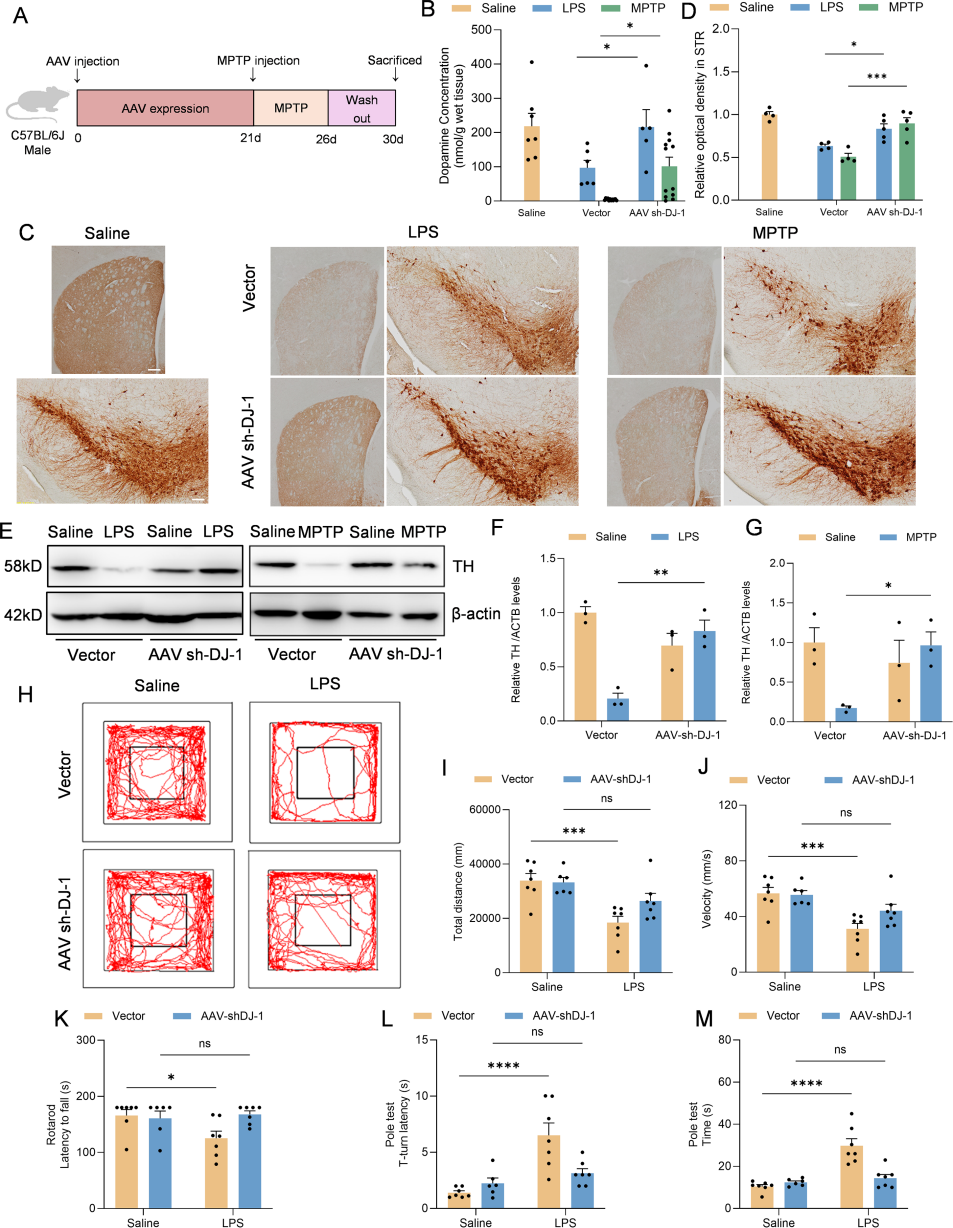
Microglia-specific DJ-1 knockdown confers neuroprotection in LPS- and MPTP-induced Parkinson’s disease models **(A)** Schematic representation of the experimental flow. C57BL/6J mice were injected with either AAV-Vector or AAV-shDJ-1 and after 21 days were treated with MPTP. **(B)** Concentration of dopamine in the SNc of mice were measured by HPLC (n ≥ 5 per group). **(C)** Immunohistochemical staining of TH-positive neurons in the SNc and TH staining for DA terminals in STR. The scale bar is 200 μm. **(D)** Quantification of relative optical density in STR. **(E)** Western blotting analysis of TH in the SNc homogenate from the indicated mice. **(F, G)** Quantification of TH protein level in the SNc homogenate supernatant of LPS **(F)** and MPTP **(G)** treated mice (n = 3 per group). **(H-J)** Representative the movement trajectories **(H)**, total distance **(I)** and statistics of the average movement speed of the LPS treated mice **(J)** during a 10-minute open field test. (**K-M)** Statistics of the time of the mice falling off the bar in the rotarod test **(K)**, the time from the start of the movement to the full head-down movement **(L)** and the time from the start of movement to the bottom of the bar **(M)** in the pole-climbing test were counted (n = 7 per group). Data were presented as mean ± SEM. Data were analysed using two-way ANOVA with Sidak’s multiple comparisons test **(B,D, F-G, I-M)**. Ns, no significance, *P<0.05, **P<0.01, ***P<0.001, ****P<0.0001.

To further demonstrate whether knockdown of microglial DJ-1 not only suppresses neuroinflammation but also protects dopaminergic neurons from inflammation-induced degeneration, we evaluated striatal dopamine content by HPLC and tyrosine hydroxylase (TH) expression in the SNc by both immunohistochemistry and Western blot in the LPS stereotactic model and the sub-acute MPTP model (**Figure 6A**). Results from both models demonstrated that DJ-1 knockdown in microglia significantly preserved striatal dopamine content (**Figure 6B**). Furthermore, the number of TH-positive neurons in the SNc, the optical density of STR (as an indicator for the density of DA terminals) as well as the TH protein expression were consistently increased after AAV-sh-DJ-1 injection followed by LPS or MPTP treatment (**Figures 6C–G**). These findings suggest that microglial DJ-1 plays a pivotal role in mediating inflammation-induced dopaminergic neuron loss, and that its depletion can confer dual benefits by reducing neuroinflammation and protecting neuronal survival indicated by the preservation of TH^+^ cells in the SNc.

Moreover, we performed behavioral assessments on the LPS-induced PD model mice. The results showed that mice with microglia-specific DJ-1 knockdown exhibited significantly increased total distance and locomotor speed in the open field test compared to control mice (**Figures 6H–J**). Additionally, their performance in both the rotarod and pole test paradigms was markedly improved, indicating better motor coordination and balance (**Figures 6K–M**). These findings further support the notion that microglial DJ-1 knockdown alleviates neuroinflammation-related motor deficits *in vivo*.

## 4 Discussion

**Figure 7:**
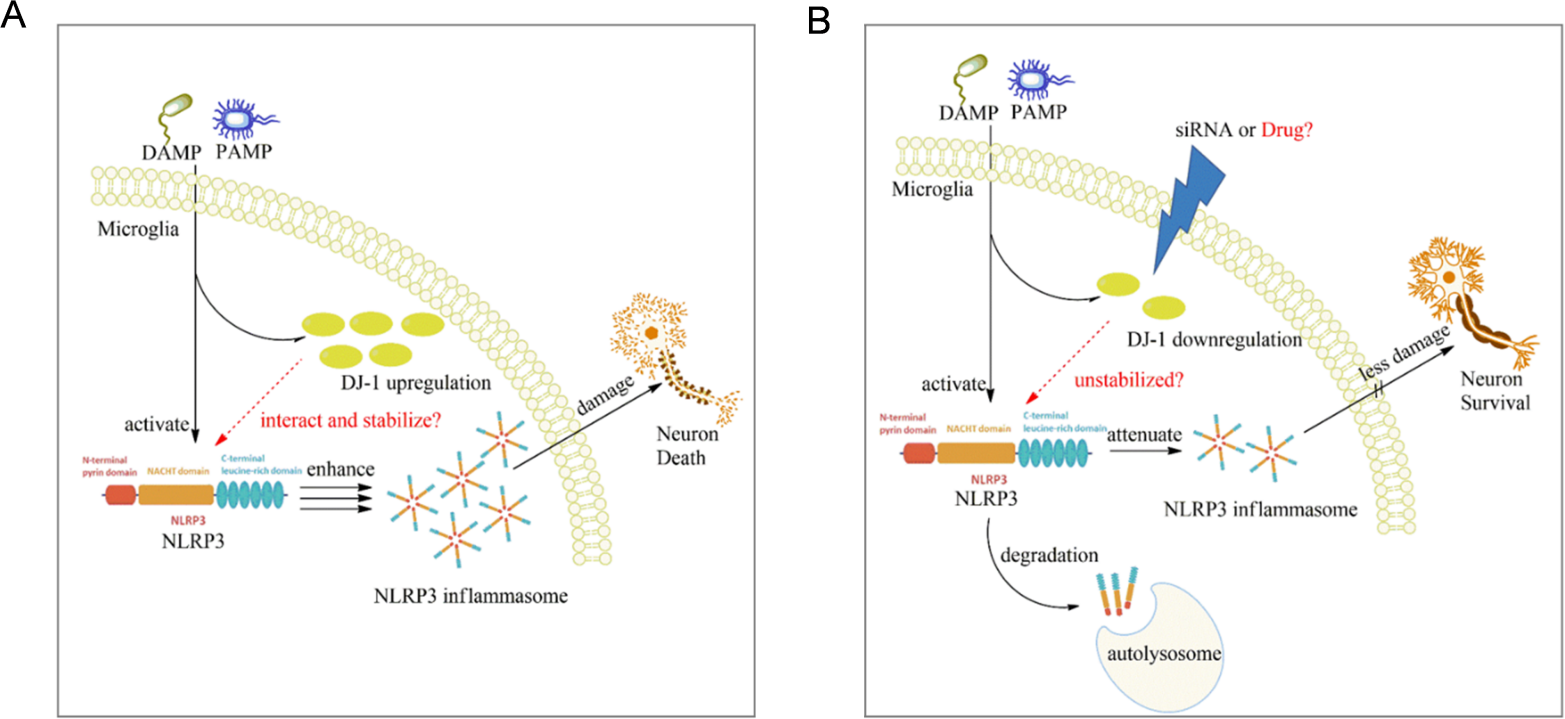
Proposed mechanism of microglial DJ-1 knockdown in suppressing NLRP3-driven inflammation. **(A)** When microglia are stimulated by exogenous PAMPs or DAMPs, the expression levels of both NLRP3 and DJ-1 are upregulated in response to the inflammatory stimuli. The increased DJ-1 helps stabilize the conformation of NLRP3, thereby ensuring the proper activation of the NLRP3 inflammasome. **(B)** In microglia with reduced DJ-1 expression due to siRNA or pharmacological intervention, the loss of DJ-1 compromises NLRP3 protein stability, making it more susceptible to degradation via the autophagy–lysosome pathway, ultimately leading to the suppression of NLRP3-mediated inflammatory responses.

This study identified DJ-1 as a previously underappreciated regulator of the NLRP3 inflammasome in microglia in the context of Parkinson’s disease. Our findings demonstrated that DJ-1 protein expression not only increased in response to inflammatory stimuli but also directly stabilized NLRP3 protein through protein-protein interactions. In microglia or BMDM, knockdown of DJ-1 led to impaired NLRP3 inflammasome activation, as evidenced by reduced ASC speck formation, caspase-1 cleavage, and IL-1β release. Moreover, this regulatory role appeared to be selective for NLRP3, as DJ-1 depletion had minimal impact on NLRC4 or AIM2 inflammasome activation. *In vivo* stereotactic injection of DJ-1-targeting microglia specific AAVs into the substantia nigra revealed diminished microglial activation, reduced NLRP3 expression, and attenuated neuroinflammatory responses following LPS challenge. These molecular changes were accompanied by preservation of TH-positive neurons and increased striatal dopamine levels, resulting in significant improvement in motor behaviors. Mechanistically, we found that DJ-1 does not affect NLRP3 transcription but instead stabilizes NLRP3 protein. By using CRISPR-Cas9-mediated DJ-1 knockout in HEK-293, further pharmacological assays using 3-MA and rapamycin suggested that DJ-1 prevents NLRP3 degradation through the autophagy-lysosome pathway (**Figures 7A, B**).

In recent years, mounting evidence has implicated chronic neuroinflammation as a major contributor to PD pathogenesis, and NLRP3 inflammasome has emerged as a central regulator of innate immune responses in the brain (30). NLRP3 inflammasome is the most abundant and functionally dominant inflammasome in microglia, compared to other inflammasomes like AIM2 or NLRC4 (9). It can be activated in microglia by multiple PD-relevant stimuli, including α-synuclein aggregates, mitochondrial dysfunction, reactive oxygen species (ROS), and environmental toxins (31). Activated microglia, through the assembly of the NLRP3 inflammasome complex, facilitates the cleavage of pro-caspase-1 and the maturation of pro-inflammatory cytokines such as IL-1β, thereby exacerbating neuronal injury (32). Thus, targeting NLRP3-mediated neuroinflammation presents a promising therapeutic avenue for slowing the disease progression in PD.

One of the strategies to inhibit NLRP3 inflammasome activation is to promote the degradation of NLRP3 through autophagy-lysosome pathway which is considered one of the major routes for NLRP3 degradation (33). Herein, we found that DJ-1 can directly bind to the NLRP3 protein and function as a stabilizer, thereby maintaining its structural integrity. Knockdown of DJ-1 promotes the degradation of NLRP3 via the autophagy-lysosome pathway. This mode of action for DJ-1 is not unprecedented, for instance, the E3 ubiquitin ligase TRIM65 has been shown to bind the NACHT domain of NLRP3 and promote its Lys48- and Lys63-linked ubiquitination and subsequent degradation via the lysosomal pathway (16). Furthermore, DJ-1 has been shown to modulate autophagy in diseases beyond the nervous system, including cancer (34, 35). In our future studies, it will be important to focus on identifying the specific domain of NLRP3 that interacts with DJ-1. Given that DJ-1 knockdown does not affect the activation of NLRC4 or AIM2 inflammasomes, despite these inflammasomes sharing structural similarities with NLRP3 (36, 37), it is reasonable to hypothesize that DJ-1 interacts with a domain unique to NLRP3. The NACHT domain is the central regulatory region responsible for the self-activation of NLR family proteins. MCC950, a selective NLRP3 inhibitor, specifically targets this domain, thereby conferring its high specificity for NLRP3 inflammasome inhibition (38). Hence, we speculated that DJ−1 most likely binds to NLRP3’s NACHT domain, or potentially the LRR domain, which is unique to NLRP3 and critical for regulating its stability and activation state. Future therapeutic strategies could focus on identifying compounds that suppress the LPS-induced upregulation of DJ-1 specifically in microglia. Such a targeted approach may effectively attenuate neuroinflammatory responses in the brain while minimizing off-target effects on neurons or astrocytes.

DJ-1 was originally identified as a recessive familial Parkinson’s disease risk gene, and mutations in DJ-1 are causatively linked to early-onset PD (39). This assertion is principally grounded in the role of DJ-1 in neurons. Dopaminergic neurons are particularly vulnerable to oxidative stress and mitochondrial dysfunction while DJ-1 could play a crucial role in protecting dopaminergic neurons by scavenging reactive oxygen species (ROS) and preserving mitochondrial function (40, 41). Loss-of-function mutations in DJ-1 compromise these neuroprotective mechanisms, rendering dopaminergic neurons more susceptible to stress-induced apoptosis and ultimately contributing to the development of PD (42, 43). While DJ-1 has been widely recognized for its neuroprotective and antioxidant functions in neurons, our study aims to investigate its non-canonical role in glial cells, particularly in the context of neuroinflammation. Given the distinct cellular functions and metabolic pathways between neurons and glial cells in the brain, DJ-1 may exert cell type-specific effects. For instance, in neurons, elevated ROS levels primarily lead to mitochondrial dysfunction and apoptosis, contributing to neurodegeneration (44, 45). In contrast, in microglia, ROS production plays an essential role in pathogen clearance and phagocytic activity (46). Therefore, as a ROS scavenger, DJ-1’s function may differ significantly depending on the cellular context, serving as a neuroprotective antioxidant in neurons while potentially modulating inflammatory signaling pathways in microglia. In this study, we specifically focused on the role of DJ-1 in microglia, aiming to investigate how it regulates the NLRP3 inflammasome, which is the most abundantly expressed and well-characterized inflammasome subtype in microglial cells.

We found that DJ−1 is upregulated in response to LPS-induced inflammatory stimulation both *in vitro* and *in vivo*, indicating a correlation between DJ−1 expression and microglial inflammatory activation. In microglia, the co-upregulation of DJ-1 and NLRP3 proteins upon inflammatory stimulation is likely mediated by the NF-κB signaling pathway. LPS activates Toll-like receptor 4 (TLR4) on microglia, which is well known to initiate downstream NF-κB signaling and promotes NLRP3 expression (47). Notably, emerging evidence also suggests that DJ-1 expression is regulated by NF-κB signaling (48, 49), indicating that both proteins may be transcriptionally induced through a common upstream pathway. Based on this, we speculated that since NLRP3 and DJ-1 are both upregulated in LPS-activated microglia, which could form part of a coordinated response to inflammatory stimulation. DJ-1 may function to stabilize the conformation of the NLRP3 protein, thereby facilitating the proper assembly and activation of the NLRP3 inflammasome and ensuring the effective initiation of downstream inflammatory signaling. A recent study reported that magnetic bead-sorted microglia from DJ-1 knockout (KO) mice, 24 hours after LPS injection, showed downregulated expression of pro-inflammatory genes such as *Ccl2*, *Ccl4*, *Il1b*, and *Nlrp3*—a pattern consistent with our findings (25). The authors proposed that chronic oxidative stress induced by DJ-1 deficiency impairs the neuroinflammatory response of microglia. Indeed, as early as 2015, it was demonstrated that DJ-1 interacts with the NADPH oxidase subunit p47^phox^ to promote ROS production in macrophages, thereby enhancing NADPH oxidase-dependent inflammatory responses (50). DJ-1 deletion resulted in significantly reduced systemic and local inflammation, as well as impaired phagocytosis and bactericidal capacity of macrophages, indicating that DJ-1-mediated oxidative stress is essential for sustaining inflammatory responses. Our study further reveals a distinct mechanism whereby DJ-1 directly interacts with and stabilizes the conformation of NLRP3, underscoring its critical role in maintaining microglial inflammatory activation.

To investigate the functional relationship between DJ-1 and the NLRP3 inflammasome in PD, we selected primary microglia as the most appropriate cellular model. Among various inflammatory stimuli, a combination of LPS priming followed by ATP stimulation has proven to be the most effective method for inducing robust NLRP3 inflammasome activation in microglia (51). In contrast, commonly used stimuli such as MPP^+^ or LPS alone fail to fully recapitulate the sequential signaling required to evaluate DJ-1’s role in inflammasome assembly and downstream inflammatory cascades (23, 24). In our study, we applied prolonged LPS priming (8 hours), which markedly elevated NLRP3 protein expression in microglia. Subsequent ATP treatment then triggered efficient inflammasome assembly and activation. Under these optimized conditions, DJ-1 knockdown enabled us to better assess its post-translational regulatory role on NLRP3 stability and function. Importantly, for the *in vivo* validation, we employed a microglia-specific AAV vector targeting DJ-1 under the control of a microglial promoter. This cell-type-specific approach allowed us to dissect the contribution of DJ-1 in microglia without affecting its expression in neurons or astrocytes. This is in contrast to previous studies using global DJ-1 knockout mice, where LPS administration exacerbated PD pathology, a result that may primarily reflect the loss of DJ-1 neuroprotective and antioxidant roles in neurons (24, 52). These seemingly contradictory findings highlight the need to consider cell-type-specific functions of DJ-1 in PD pathology. Furthermore, seemingly opposite outcomes reported for DJ−1 in other inflammatory models can be rationalized by the nature and intensity of the inflammatory trigger. For example, in a traumatic spinal cord injury (tSCI) model, DJ−1 suppresses NLRP3 activation via the SOCS1/Rac1/ROS pathway (22). However, tSCI elicits neuroinflammation primarily through mechanical tissue disruption, and the resulting NLRP3 activation is relatively modest and slow compared with the potent, TLR4−driven response induced by LPS (53, 54). Likewise, in the MPTP model, where mitochondrial toxicity in dopaminergic neurons is the primary insult, microglial NLRP3 activation is largely a secondary reaction to extensive neuronal death (55, 56).

A limitation of the present study is that we did not directly assess the effects of DJ-1 deficiency on microglial mitochondrial homeostasis. Given that mitochondrial function is crucial for regulating microglial inflammatory activation (57, 58), future studies are needed to investigate how loss of microglial DJ-1 impacts mitochondrial dynamics, ROS handling, and energy metabolism in the context of inflammation. Another limitation is that we did not examine the impact of microglial DJ-1 deficiency on α-synuclein–related pathology, a key driver of PD progression. Therefore, our current findings mainly reflect the role of DJ-1 in neuroinflammation (LPS) and oxidative stress (MPTP), but do not fully address its contribution to protein aggregation–driven PD pathology. Future studies could employ A30P transgenic mice or injection of α-synuclein preformed fibrils (PFFs) combined with microglial DJ-1 knockdown to explore whether DJ-1 loss modulates α-synuclein–related pathological progression.

In summary, our study reveals that DJ-1 directly interacts with the NLRP3 protein and protects it from degradation via the autophagy-lysosome pathway. Both *in vivo* and *in vitro* experiments demonstrated that DJ-1 knockdown in microglia suppresses NLRP3 inflammasome activation, thereby alleviating neuronal injury and apoptosis. These findings suggest that microglial DJ-1 may serve as a potential therapeutic target for the development of NLRP3 inhibitors, ultimately mitigating neuroinflammation and slowing the progression of Parkinson’s disease.

## Data Availability

The original contributions presented in the study are publicly available. This data can be found here: https://zenodo.org/records/17033984.

## Supplementary material

Supplementary Figure 1 DJ-1 was significantly downregulated after siRNA administration in primary microglia and BMDM. **(A, B)** Western blotting analysis of DJ-1 in microglia **(A)** and BMDM (**B–D)** Quantification of DJ-1 in microglia **(C)** and BMDM (D) (n ≥ 3 per group). Data were presented as mean ± SEM. Data were analysed using two-tailed Student’s t-test **(C, D)**. ***P* < 0.01, ****P* < 0.001.
