# Supplementary figures and images for: Loss of DJ-1 alleviates microglia-mediated neuroinflammation in Parkinson’s disease via autophagy-lysosomal degradation of NLRP3
Source: Front Immunol. 2025 Sep 8;16:1656729. doi: 10.3389/fimmu.2025.1656729 (PMC12450706; doi:10.3389/fimmu.2025.1656729)

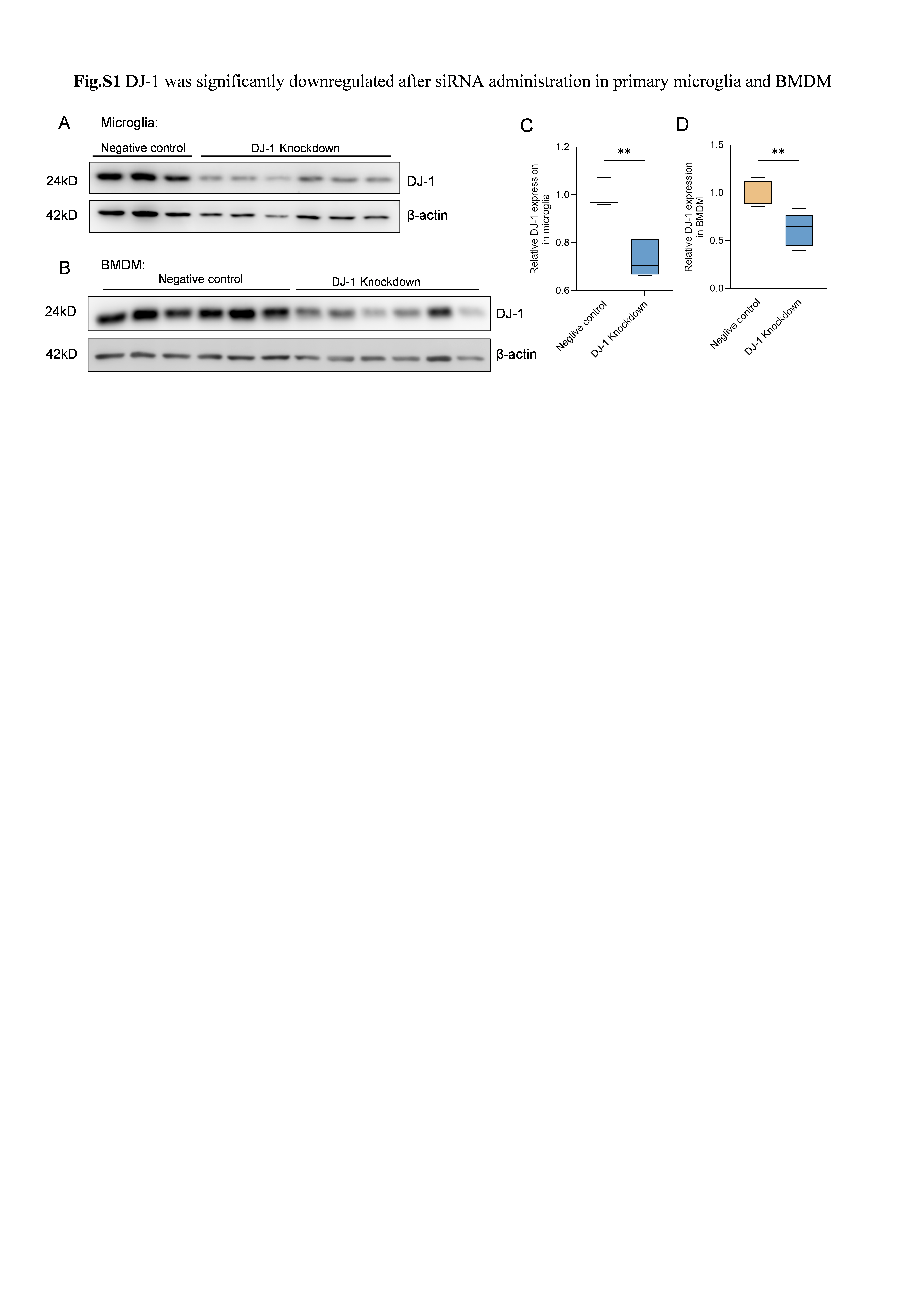

Supplement: Supplementary Figure 1 — DJ-1 was significantly downregulated after siRNA administration in primary microglia and BMDM. (A, B) Western blotting analysis of DJ-1 in microglia (A) and BMDM (B–D) Quantification of DJ-1 in microglia (C) and BMDM (D) (n ≥ 3 per group). Data were presented as mean ± SEM. Data were analysed using two-tailed Student’s t-test (C, D). **P < 0.01, ***P < 0.001. [file Image1.tif]
